# Abusive advertising of food and drink products on Brazilian television

**DOI:** 10.1093/heapro/daab025

**Published:** 2021-07-19

**Authors:** Julia S Guimarães, Laís A Mais, Fernanda H M. Leite, Paula M Horta, Marina O Santana, Ana P B Martins, Rafael M Claro

**Affiliations:** 1 Federal University of Minas Gerais (Universidade Federal de Minas Gerais—UFMG), Belo Horizonte, MG, Brazil; 2 Brazilian Institute for Consumer Defense (Instituto Brasileiro de Defesa do Consumidor—Idec), Brazil

**Keywords:** food, advertising, television, non communicable diseases

## Abstract

In Brazil, any advertising strategies that take advantage of the child’s judgment or induce consumers to make harmful health choices are considered abusive. However, the efficacy of restrictions on their use on television (TV) food advertising remains poorly understood. This study analysed the extent and nature of abusive techniques according to national regulations and patterns in their use in food-related ads. Cross-sectional studyrecorded the programming of the three most popular TV channels (6 am–12 am), during eight non-consecutive days, in April 2018. Data collection was based on the INFORMAS protocol and two national regulations. Descriptive analyses were used to describe the types of food ads and abusive techniques used in ultra-processed products (UPP) ads. Principal component analysis was applied to identify patterns of abusive marketing techniques and to relate them to specific food groups. More than 90% of food ads included at least one UPP. Overall, 10.1% of UPP ads targeted children and 57.7% used some kind of abusive technique directed to children and adolescents. Most ads contained messages inducing harmful health choices did not present adequate and clear information about the product and contained more than one type of abusive technique. Four out of five patterns in the use of abusive techniques in UPP ads were specifically directed to children, and fast-food meals were associated with three out of five patterns. The high abusiveness of food advertising in UPP ads should be considered a public health concern given their impact on children’s food choices and health.

## INTRODUCTION

Childhood obesity is associated with a higher chance of overweight and obesity in adulthood (Park *et al*., 2012) as well as a greater risk to develop non-communicable diseases (NCDs) (Abdullah *et al*., 2011; Park *et al*., 2012). Although childhood obesity rates are higher in upper-middle-income countries, the number of children with excess weight is increasing at fastest pace in lower-middle-income countries (UNICEF and WHO, 2018). An important contributor to this condition is the influence of food marketing on food choices (Cairns *et al*., 2013; Kelly *et al*., 2015; Buchanan *et al*., 2018). Additionally, previous studies that analysed food marketing on Brazilian television (TV) (Maia *et al*., 2017) and worldwide (Rincón-Gallardo Patiño *et al*., 2016; Vandevijvere *et al*., 2017; Allemandi *et al*., 2018) showed that the majority of advertisements (ads) promoted energy-dense and nutrient-poor food items. Thus, it is essential to have policy actions to regulate food marketing, especially those directed to children, and it is recommended to combat obesity and NCDs by international health organizations (WHO, 2010, 2013).

The Consumer Defense Code (*Código de Defesa do Consumidor—*CDC) is a Brazilian law responsible for protecting consumers against all kinds of abusive advertising. It classifies and determines the principles of advertising and states that abusive and misleading advertisements cannot be promoted (Consumer Defense Code, 1990). The CDC considers abusive any advertising or marketing communication techniques that takes advantage of the child’s judgment and experience, or induces consumers to make harmful health choices (§2, article 37 of the Law 8078, 1990) (Consumer Defense Code, 1990). Furthermore, the National Council of Children and Adolescents’ Rights (*Conselho Nacional dos Direitos da Criança e do Adolescente—*Conanda) established the Resolution no. 163, which regulates abusive aspects of advertising directed to children (Resolution No. 163, 2014). This resolution specifies which kind of marketing techniques are considered abusive for children and adolescents and, in its article 3, specifically directs restrictions to protect adolescents against abusive advertising (Resolution No. 163, 2014). Still, in the current Brazilian marketing scenario, there is a significant exposure to unhealthy advertising. Thus, to better understand abusive aspects of advertising, it is imperative to monitor population exposure to unhealthy food promotions, especially in mass communication channels (such as TV), and for the establishment of appropriate measures related to this issue (Kelly *et al.*, 2013). Therefore, this study aimed to analyse the extent and nature of abusive advertising techniques according to the CDC and the Conanda’s Resolution and to determine patterns in the use of them in food and drink ads on the three major Brazilian free-to-air TV channels.

## MATERIALS

### Sample, data collection and coding

This was a cross-sectional study, which recorded programs and ads aired on the three most popular free-to-air TV channels in Brazil (*Rede Globo*, *Record* and *SBT*), according to data from Kantar-Ibope (*Instituto Brasileiro de Opinião Pública e Estatística—*IBOPE) (IBOPE, 2018) , during eight non-consecutive days (four weekdays and four weekend days) from April 1 to 30 2018 between 6 am and12 am each day, for a total time of 144 h per channel and 432 h overall.

Advertisement (ad) information was extracted through a digital questionnaire based on the International Network for Food and Obesity/Non-Communicable Diseases (NCDs) Research, Monitoring and Action Support (INFORMAS) protocol (Kelly, 2017) (Epi Info^®^ software, version 7.2.2.6). In the current study, the following information was investigated for each ad: country name, region of collection, collection year, channel, date of recording and day of the week or weekend. Ads were classified according to the INFORMAS protocol and categorized into two main groups: food-related group and non-food-related group (Kelly, 2017). For food-related ads, more details were collected as follows: brand/company name, product name and description, food category (according to the NOVA classification system, more details presented ahead), targeted audience and the use of abusive advertising techniques.

Data coding was independently conducted by two trained researchers and the results and/or any divergence compared on a regular basis. All datasets passed through three crosschecks to standardize all values and correct any data collection/entering error. The inter-coder reliability was calculated at the beginning of the data collection with a 10% subsample. The percentage of agreement was high, ranging from 90.4% to 99.7% (Kelly, 2017).

### Classification of advertised foods: The NOVA classification system

The NOVA classification system categorizes foods and beverages according to the industrial processing level involved in their production into four groups: unprocessed or minimally processed foods, processed culinary ingredients, processed foods and ultra-processed food products (UPP) (Monteiro *et al*., 2010, 2019).

Once data extraction was concluded, food-related ads were classified according to the NOVA classification system, only considering eligible food or drink products. Any other items not related to food or drink products were excluded from the analyses. Also, ads of restaurants, fast-food chains or supermarkets, as well as food-related ads announcing more than one product, passed through a ‘less healthy’ product selection method. In this method, all foods and beverages were listed, and according to the NOVA classification system (Monteiro *et al*., 2019), the ‘less healthy’option (based on the level of industrial processing) was chosen to be classified. If products were in the same NOVA category (e.g. processed foods or UPP), the selection of the ‘less healthy’ was made according to the nutrition facts panel from the product label. In that case, the total amount of critical nutrients, such as total fat, saturated fat, sodium and sugar per 100 g/ml was compared, and the ‘less healthy’ option was the one with overall higher amount of critical nutrients.

### Abusive advertising

The use of abusive marketing techniques in food or drink ads was assessed according to two major Brazilian regulations: the CDC and the Conanda’s Resolution no. 163. Chart 1 shows the abusive techniques assessed in the current survey. According to the regulations and for the purpose of this study, it was considered children every person until 12 years of age and adolescentand every person from 12until 18 years of age.

**Chart 1:** Abusive advertising techniques according to the Consumer Defense Code (CDC) and the Resolution no.163 of the National Council of Children and Adolescents’Rights (Conanda)

Abusive techniques	
CDC	
Techniques that induce consumers to make harmful health choices: Message that stimulates excessive consumption of UPP^a^ Promotions or free gifts that stimulate excessive consumption of UPP Images alluding to excessive consumption of UPP^b^ Other technique that induces unhealthy food choices^c^	Absence of adequate and clear information such as:^d^ Specification of the product’s ingredients Specification of the product’s quantity Specification of the product’s price Clarification on the risks associated with excessive consumption Product’s nutritional information (e.g. amounts of sugar, sodium, fat and calories)
Conanda’s Resolution no. 163	
Abusive techniques directed to children and adolescents: Children’s language Children’s songs or songs sung by children Image of children^e^ Presence of celebrities appealing to children Children’s characters or presenters Cartoon or animation Toys^f^ Prizes or free gifts Competitions or games^g^ Use of special effects Excessive colors^h^ Other abusive technique directed to children^i^	Techniques that violate general principles of the Resolution no. 163 (article 3): Encourages teenagers to embarrass their guardians Promotes offense or discrimination Induces a feeling of inferiority (if the product/service is not consumed) Incites illegal activities Encourages environmental degradation Misleading information about the product, its techniques and functioning^j^

UPP: ultra-processed food products.

a If food ads announced any message stimulating excessive intake of UPP (e.g. ‘eat at any time’, ‘eat all the time’).

b If the ad showed people consuming UPP and feeling good/pleasant, or presented images of UPP linked with other factors such as low price.

c When the ad stimulated other unhealthy behaviors, such as excessive consumption of dietary supplements or unhealthy food products that was not classified as ultra-processed foods according to the NOVA classification system.

d It was considered abusive any ad that did not present those information.

e Presence of any children, including in the form of cartoon or animation.

f Presence of toys, board games, card games or video games.

g Presence of any type of competition or game (not listed on *toys*), including advergames, in which consumers could participate or even if that happened only in the advertising dimension.

h When bright colors were used to attract consumers’ attention or original colors were altered to match with colors from a specific brand.

i If any other abusive technique was used in the ad (e.g. reference to a follow up of the ad on social media, the presence of a catching song not sung by children, or any reference to events appealing to children or adolescents).

j The ad presented misleading information about the advertised product (e.g. hiding information related to the product’s packaging, size or appearance).

The current paper focused specifically on the techniques used in UPP ads. In order to investigate the presence of abusive techniques in food and drink ads, information regarding the CDC was collected for all UPP ads, whereas information on abusive techniques according to the Conanda’s Resolution was only collected for food or drink ads targeting children, adolescents or general audience, which included any marketing tactics that appeals to children or adolescents.

### Statisticalanalysis

Absolute and relative frequencies and 95% confidence interval (95% CI) described the number and type of ads broadcasted on Brazilian TV (according to the NOVA classification system), and the main types and number of abusive techniques used in UPP ads. Further, the principal component analysis (PCA) was applied (polychoric correlation matrix) to investigate the behavior of abusive advertising techniques used in UPP ads. Low frequency techniques (≤5%) were not included. Components (from now on mentioned as patterns) were retained based on Kaiser’s criteria (Eigenvalue ≥ 1) and scree plot analysis. Component scores (one for each retained pattern) were predicted and saved. The association between these scores and the subgroups of UPP was analysed through linear regression models. The score was used as the dependent variable, while the UPP subgroups (a set of dummy variables) were introduced as independent ones. Regression residuals were analysed to verify the presence of homoscedasticity. All statistical analyses were conducted using the Stata statistical software package (version 14.2). Any difference in the values was considered statistically significant when the 95% CI did not overlap or when the *p*value was equal or lower than 0.05 (*p* ≤ 0.05).

## RESULTS

During the 432 h of TV recording, 1156 food-related ads were identified at an average rate of 0.89 ads per channel per hour (ads/channel/hour). From that, 298 were excluded from the analyses because they were not considered a specific food or drink item (e.g. 218 ads were dietary supplements, 59 food or drink retailer not depicting a food or drink product and 21 food or drink company or brand without food or drink product). Only 858 food and drink product ads were classified according to the NOVA classification system. From all food and drink product ads, 90.8% included at least one UPP at an average rate of 0.60 ads/channel/hour (Table 1).

**Table 1:** Frequency of food and drink advertisements (ads) according to the NOVA classification system, April 2018 (*n* = 858)

Food category	*n*	%	CI 95%
Unprocessed or minimally processed foods	67	7.6	5.9–9.7
Processed culinary ingredients	7	1.0	0.4–2.0
Processed foods	4	0.6	0.2–1.6
Ultra-processed food and drink products	780	90.8	88.5–92.6
*Soft drinks*	*246*	*28.9*	*25.8*–*32.2*
*Alcoholic beverages*	*133*	*14.2*	*12.0*–*16.8*
*Fast-food meals*	*109*	*13.8*	*11.5*–*16.5*
*Nuggets and other ultra-processed meat products*	*83*	*10.0*	*8.0*–*12.4*
*Icecream, chocolate and candies*	*57*	*6.6*	*5.0*–*8.5*
*Other sweetened beverages*	*53*	*5.7*	*4.3*–*7.6*
*Pastries, cakes, and cookies*	*37*	*4.4*	*3.2*–*6.2*
*Margarine*	*35*	*4.1*	*2.6*–*5.2*
*Sauce*	*15*	*1.7*	*1.1*–*3.1*
*Savory packaged snacks*	*8*	*0.9*	*0.5*–*2.1*
*Breakfast cereals*	*2*	*0.2*	*0.1*–*1.2*
*Ready-to-heat meals*	*2*	*0.2*	*0.0*–*1.0*
*Total*	*780*	*90.8*	*88.5*–*92.6*
Total	858	100.0	

CI: confidence interval.

Overall, 10.1% of UPP ads targeted children, 14.5% adults and 75.4% targeted the general audience. More than half (57.7%) of UPP ads used some kind of abusive technique directed to children and adolescents, the most used one was excessive colors to attract consumers’ attention (45.7%). Further, 97.6% of UPP ads contained messages inducing consumers to make harmful choices for health. Among these, 92.5% used images alluding to excessive consumption of UPP and 22.6% contained promotions or free gifts that stimulated excessive consumption of UPP. And 89.6% of UPP ads did not present adequate and clear information about the product (Table 2).

**Table 2:** Detailed abusive advertising techniques, according to the Consumer Defense Code (CDC) and the Resolution no.163 of the National Council of Children and Adolescents’ Rights (Conanda), use dinultra-processed food products (UPP) advertisements (ads), April 2018 (*n* = 780).

Abusive techniques	%	CI 95%
*CDC*		
**Techniques that induce consumers to make harmful choices for health**	**97.6**	**96.4–98.8**
Images alluding to excessive consumption of UPP	92.5	90.5–94.5
Promotions or free gifts that stimulate excessive consumption of UPP	22.6	19.4–25.8
Other technique that induces unhealthy food choices	1.9	0.8–2.9
Message that stimulates excessive consumption of UPP	1.0	0.2–1.7
**Absence of adequate and clear information**	**89.6**	**87.2–91.9**
*Conanda’s resolution*		
**Abusive techniques directed to children and adolescents**	**57.7**	**53.9–61.4**
Excessive colors	45.7	42.0–49.5
Other abusive techniques directed to children or adolescents	16.1	13.4–18.8
Children's characters or presenters	14.1	11.5–16.8
Cartoon or animation	14.1	11.5–16.8
Prizes or free gifts	13.6	11.0–16.2
Image of children	12.9	10.4–15.4
Presence of celebrities appealing to children	11.4	9.1–13.7
Children’s language	8.0	5.9–10.0
Toys	6.0	4.1–7.8
Children’s songs or songs sung by children	3.8	2.4–5.3
Use of special effects	3.0	1.6–4.3
Competitions or games	0.0	–
**Techniques that violate general principles of the Conanda’s resolution**	**6.9**	**5.0**–**8.9**
Promotes offence or discrimination	4.0	2.6–5.4
Misleading information about the product, its techniques and functioning	1.8	0.8–2.9
Induces a feeling of inferiority (if the product/service is not consumed)	1.1	0.3–1.8
Encourages teenagers to embarrass their guardians	0.0	–
Incites illegal activities	0.0	–
Encourages environmental degradation	0.0	–

CI: confidence interval; UPP: ultra-processed food products.

We found that most ads promoting UPP (88.4%) contained more than one type of abusive technique. Half of them (50.2%) combined abusive techniques directed to children and adolescents with messages encouraging harmful health choices as well as the absence of adequate and clear information. The use of messages encouraging harmful health choices along with the absence of adequate and clear information was observed in 30.4% of ads (Table 3).

**Table 3:** Frequency of types of abusive techniques used inultra-processed food products (UPP) advertise ments (ads) classified according to the NOVA classification system, April 2018 (*n* = 780).

Abusive techniques	%	CI 95%
Ultra-processed food products		
A + C + D	50.2	46.5–54.0
C + D	30.4	27.0–33.9
C	9.6	7.5–12.1
A + B + C + D	6.6	5.0–8.6
D	2.0	1.2–3.5
A + C	0.8	0.3–2.0
B + D	0.3	0.1–1.3
Total	100.0	

CI: confidence interval; A: abusive techniques directed to children and adolescents; B: techniques that violate general principles of article 3 of the Conanda’s Resolution; C: abusive techniques that induce consumers to make harmful health choices; D: absence of adequate and clear information about the product.

In order to analyse patterns in the use of abusive techniques in food advertising in UPP ads, the PCA was conducted with every technique, presented in Chart 1, that had a frequency of at least 5.0% among UPP ads (Table 2). Such analysis was able to predict 61.2% of the variability. We identified five patterns (Table 4), of which four were specifically directed to children or adolescents. Each pattern was named according to its main characteristic (highlighted in bold), as follows: Pattern 1—‘Childish’, Pattern 2—‘Prizes’, Pattern 3—‘Child celebrity’, Pattern 4—‘Unclear’ and Pattern 5—‘UPP to children’. The first pattern was mostly composed of children’s characters or presenters (0.5803), cartoons or animations (0.5803), children’s language (0.4686) and the presence of toys (0.2967). The second one, ‘Prizes’, was mostly characterized by the use of prizes and free gifts (0.6177), promotions or free gifts that stimulated excessive consumption of UPP (0.5138), excessive colors (0.4954) and the presence of toys (0.2943). The ‘Child celebrity’ pattern contained celebrity known to appeal to children (0.6743) and other abusive techniques directed to children (0.6811). The fourth pattern, ‘Unclear’, had negative association with the use of images alluding to excessive consumption of UPP (−0.3113) and promotions or free gifts that stimulate high consumption of UPP (−0.4384) and a positive association with to the absence of adequate and clear information (0.7824). The last, but not the least, pattern was mostly composed of the presence of children (0.6061), use of images alluding to excessive consumption of UPP (0.6060) and excessive colors (0.4067).

**Table 4:** Factor loadings of abusive techniques used in ultra-processed food products (UPP) advertisements (ads), according to the NOVA classification system, of PCA. April 2018 (n = 780)

Variables	Patterns				
	Childish	Prizes	Child celebrity	Unclear	UPP to children
Children’s language	0.4686	0.0903	0.0146	−0.0111	0.1719
Image of a child	0.1005	−0.0320	−0.1913	0.1754	**0.6061**
Presence of celebrities appealing to children	0.0224	−0.0223	**0.6743**	0.0096	−0.0131
Children's characters or presenters	**0.5803**	−0.0627	0.0078	−0.0189	−0.0159
Cartoon or animation	**0.5803**	−0.0627	0.0078	−0.0189	−0.0159
Toys	**0.2967**	**0.2943**	0.0152	0.0712	−0.2320
Prizes or free gifts	−0.0204	**0.6177**	−0.0771	0.0615	−0.1085
Excessive colors	−0.0708	**0.4954**	0.1640	0.2413	**0.4067**
Other abusive techniques directed to children or adolescents	−0.0035	0.0011	**0.6811**	−0.0001	−0.0142
Promotions or free gifts that stimulate excessive consumption of UPP	−0.0142	**0.5138**	−0.0487	**−0.4384**	−0.0594
Images alluding to excessive consumption of UPP	−0.0472	−0.0547	0.0953	−**0.3113**	**0.6060**
Absence of adequate and clear information	−0.0184	0.0436	0.0000	**0.7824**	−0.0220

UPP: ultra-processed food products; values in bold are dominant variables (represent correlation greater than 0.25) in PCA’s pattern.

The results from the linear regression models (Table 5) demonstrate that the ‘Childish’ pattern was associated with fast-food meals (β = 1.7109; *p* < 0.01) and ultra-processed meat products (β = 1.4085; *p* < 0.01); while the ‘Prizes’ pattern was highly associated with fast-food meals (β = 1.9183; *p* < 0.01) and the ‘Child celebrity’ pattern with margarine (β = 2.6280; *p* < 0.01). The ‘Unclear’ pattern, on the other hand, was not associated with any of UPP ads, suggesting that the abusive techniques related to it were distributed among all food and drink products since its frequency was considered high (89.57%). Thus, although no positive association was found with a specific food product, the values were significant (*p* < 0.05). Finally, the ‘UPP to children’ pattern was highly associated with both soft drinks (β = 1.5274; *p* < 0.05) and other sweetened beverages (β = 1.5017; *p* < 0.05) and with ice-cream, chocolate and candies (β = 1.1866; *p* < 0.05) at lower level.

**Table 5:** Linear regression analyses of ultra-processed food products (UPP), according to the NOVA classification system, presented in each pattern of the PCA, April 2018 (*n* = 780).

Food and drink products	Patterns									
	Childish		Prizes		Child celebrity		Unclear		UPP to children	
	β	p	β	p	β	p	β	p	β	p
Pastries, cakes and cookies	NS		0.5113	0.030	NS		−2.0839	<0.001	0.6253	0.001
Icecream, chocolate and candies	NS		NS		NS		−2.8879	<0.001	1.1866	<0.001
Savory packaged snacks	NS		NS		NS		−2.7739	<0.001	−0.7770	0.026
Soft drinks	0.5055	<0.001	NS		0.7289	0.0000	−2.7342	<0.001	1.5274	<0.001
Other sweetened beverages	1.0945	<0.001	−1.0138	<0.001	−0.5785	0.0040	−2.8788	<0.001	1,5017	<0.001
Nuggets and other ultra-processed meat products	1.4085	<0.001	−0.4838	0.002	−0.3121	0.0600	−1.7605	<0.001	−0,4258	<0.001
Ready-to-heat meals	NS		NS		NS		−2.8968	0.001	NS	
Sauce	NS		NS		NS		−2.9145	<0.001	−1.1284	<0.001
Breakfast cereals	NS		NS		NS		−2.4052	0.007	NS	
Margarine	NS		NS		2.6280	0.0000	−2.4991	<0.001	NS	
Alcoholic beverages	NS		−0.4130	0.001	−0.3899	0.0050	−2.2129	<0.001	NS	
Fast-food meals	1.7109	<0.001	1.9183	<0.001	NS		−1.2391	<0.001	NS	

UPP: ultra-processed food products; *p*: *p*-value; NS: statistically non-significant (*p* > 0.05); β: coefficient from the linear regression.

## DISCUSSION

This is the first research to investigate the use of abusive techniques in food and drink ads aired on Brazilian free-to-air TV in light of the country`s regulations. Currently, little is known or monitored about the abusiveness of food advertising worldwide. As far as we are aware, most of the studies that analysed food advertising on TV focused their attention on advertising strategies (Gómez *et al*., 2017; León-Flández *et al*., 2018; Kent *et al*., 2011) rather than assessing abusive characteristics regarding current regulations. Even research from other countries that applied the INFORMAS protocol (Kelly *et al*., 2015; Allemandi *et al*., 2018) did not approach the topic of abusive and misleading ads. Further, this is the first paper to analyse patterns in the use of abusive tactics and the main food groups associated with each pattern, emphasizing both the relevance and innovation of this study.

Our results show that, although food-related ads represented only 14.1% of all Brazilian free-to-air TV advertising, at least one UPP was shown in 90.8% of all food and drink ads. Not only the exposure to unhealthy food products was high, but also the nature of UPP ads was highly associated with the use of abusive techniques by advertisers. We found that six out of 10 UPP ads (57.7%) directed abusive tactics to children and adolescents and that almost all ads (98.6%) did not present adequate and clear information about the product, regardless of the combination with other abusive techniques. On top of that, almost all UPP ads (97.6%) induced consumers to make harmful choices for their health.

The power of advertising over food choices is already well known. A systematic review (18 articles from 2000 to 2014) showed positive association between food advertising exposure and food choices in adults (Vukmirovic, 2015). However, reliable evidence shows that children are more susceptible to advertising than adults. The results from another systematic review revealed that an acute exposure to food advertising (such as a 5-min advergame or a 14-min cartoon) increased food intake among children but not in adults (Boyland *et al*., 2016). However, an experimental study, conducted in three countries with almost 3000 participants aged 8–21, showed that unhealthy food advertising can stimulate unhealthy food intake both in adults and children (Giese *et al*., 2015).

Additionally, a recent experimental study, performed with 624 preschoolers (3–5 years old), showed that the effects from the exposure of high-sugar breakfast cereals (SBCs) ads can remain for over seven days (Emond *et al*., 2019). In the study, parents assessed the ad exposure (on daily TV watching) and child intake of SBCs during the previous seven days of the follow-up (every eight weeks, for a year). And they observed that children with higher exposure to SBCs ads had an increased risk of SBCs intake (Emond *et al*., 2019).

According to the CDC, advertisements should be easily identifiable as a marketing piece; otherwise, it should not be broadcasted. Besides, this law emphasizes that any kind of advertising that takes advantage of children’s lack of maturity or experience is abusive and therefore should not be promoted (Consumer Defense Code, 1990). Studies demonstrate that children begin to differentiate ads from programming at the age of five years (Lapierre *et al*., 2017), but until they are seven or eight, they cannot understand advertising intents to convince consumers to buy the advertised product (Lawlor and Prothero, 2013; Lapierre *et al*., 2017). However, an experimental study, conducted with 100 children from 3to 10 years old, identified that only when children are between 7and 10, they have a medium-high brand recognition (Vecchio *et al*., 2014). Moreover, another experimental study conducted with 130 children (from 3to 9years old) showed that only older children (8–9 years old) could detect and explain distorted claims in ads (Mills and Elashi, 2014). Therefore, if children are not able to identify the ad as a marketing piece and cannot identify it as misleading, then the advertising takes advantage of their lack of cognitive development, experience and judgment ability. Thus, ads with any abusive techniques directed to children (more than half of all UPP ads according to our findings) disrespects the CDC, therefore, should not be marketed. Additionally, the Conanda’s Resolution no. 163 determines (in its article 2) that an ad is abusive if it uses any of the techniques presented on Chart 1 (Resolution No. 163, 2014). Thus, 57.7% of all UPP ads identified in our study would be considered eligible for restrictions according to existing regulation, given that the CDC prohibits any abusive advertising and the Conanda’s Resolution no. 163 explicitly expresses the concept of abusiveness of ads aimed at children and adolescents.

In addition, the CDC classifies as abusive all advertising strategies that induce consumers to make harmful choices for their health or safety (Consumer Defense Code, 1990). According to the scientific literature, the relationship between UPP and metabolic syndrome, obesity and NCDs is well-established (Tavares *et al*., 2012; Canella *et al*., 2014), emphasizing that, overall, the frequent consumption of UPP is harmful for the population’s health. Our research identified that 97.6% of UPP ads induced consumers to make harmful choices for health. In such way, it is understandable that by doing so, those ads were opposing to what is determined by current regulations.

Additionally, to better discuss the abusive techniques used in the ads, we have identified specific patterns in the use of abusive features in Brazilian food advertising. We found that four out of five patterns were directed towards children or adolescents and that fast-food meals played an important influence in three out of the five patterns identified by the PCA. The definition of the major characteristics associated with specific food groups also helped us to better understand the nature of Brazilian food and drink advertising.

Our findings demonstrate the urgent need to measure, monitor and tackle the use of abusive techniques in food advertising. One important way to combat the misleading persuasive features of unhealthy food marketing is through education and population’s empowerment (especially children) regarding the nature of abusive characteristics used in food ads, and the harmful effects associated with excessive consumption of UPP (Resolution No. 163, 2014; van Dam and Reijmersdal, 2019). However, children’s vulnerability appears to be more associated with their own cognitive development rather than their knowledge of advertising concepts, suggesting that the educational way is not enough to solve the problem (Lapierre, 2015). It is also imperative to reduce children’s exposure to unhealthy food ads in order to prevent eating behaviors known to follow that practice (Giese *et al*., 2015; Lapierre, 2015). In Brazil, both children and teenagers (from 4 to 17 years old) watch TV for an average of 5 h and 35 min per day (Criança e Consumo, 2015). Further, only one of the channels analysed in this study (*SBT*) has a specific period of the day dedicated to children in the morning and a small portion (5.2%) in the evening (Galhardi *et al*., 2015). The other two channels (*Rede Globo* and *Record*) have either very little programming to children or none (Galhardi *et al*., 2015), which means that children and adolescents could be watching TV at any time of the day. Given that UPP ads were shown at an average rate of 0.60 ads/channel/hour, we could conclude that an average Brazilian child would be exposed to approximately 1222 UPP ads per year. Besides, considering the average time of TV watching constant from the age of 4–17 years old, until the end of a child’s youth, he/she would be exposed to 15 886 UPP ads.

The exposure of the Brazilian population to unhealthy food advertising is significantly high, which could be the result of the lack of enforcement of current legislation. The National Council of Self-Regulation of Advertising (*Conselho Nacional de Autorregulamentação Publicitária—*CONAR) is the agency responsible to judge cases of abusive advertising in Brazil through a self-regulatory system. However, evidence shows that self-regulatory systems are not enough to protect consumers against unhealthy food marketing and many countries that have implemented this type of advertising control system face difficulties (Kent *et al*., 2011; Busse and Bernabé-Ortiz, 2018). Further, because the CONAR is not a government agency, its decisions are only seen as recommendations for members of the council, which could choose to follow or not (CONAR. Conselho Nacional de Autorregulamentação Publicitária, 2013).

Thus, in Brazil, there are two systems of advertising regulations: industry self-regulation and statutory legislation. But, for the Brazilian regulatory system to really be effective, there are three major steps proposed by the World Health Organization (WHO) to be defined in order to help address the limitations from the self-regulatory and statutory actions (WHO, 2012; Boyland and Harris, 2017). First, to delimit the age range for children who should be protected from unhealthy food marketing and to establish what types of marketing should not be used to promote UPP (WHO, 2012; Correa *et al*., 2019). Another important point is to judge the content of an ad before it is transmitted. Currently, in Brazil, an abusive ad is only removed from the media if there is a formal complaint and if the regulatory agencies determine that there was an irregularity. But when the ad is removed, people were already exposed to the inappropriate content. On the contrary, the exposure could really be prevented, once the ad, which content violates the regulations, is prohibited before even being released. For this reason, we propose a higher involvement of the judiciary branch on this issue. It is demanded to enable and raise awareness about this problem so legal agents could identify, analyse, denounce and punish wrongdoing advertising, especially for food and drink products.

Another concern relies on the fact that the current system implies subjectivity since no definitive definitions for what is considered abusive advertising, or criteria to classify food and drink products to be targeted for marketing restrictions, are presented. These issues could be solved through the use of a Nutrient Profile Models (NPM), as those proposed by the Pan American Health Organization (PAHO) (PAHO, 2016) or the World Health Organization Regional Office for Europe (WHO/Europe) (WHO, 2015) NPM were proven as strong tools to classify food and drink ads (Rincón-Gallardo Patiño *et al*., 2016) . Their use also has the advantage of removing primary focus on targeted audience—which is not necessarily simple to measure and identify (since ads tend to search for the broadest audience possible in order to maximize their impact and most products can be consumed by individuals from all ages) and can also mask the fact that children might also be influenced by ads directed at adults. Finally, even though WHO’s recommendations are directed to children, one should keep in mind that adolescents and adults are also influenced by such exposure, once again indicating that regulatory focus should be on products instead of age groups.

## CONCLUSION

This study investigated the extent and nature of abusive techniques in food and drink advertising on the three most popular free-to-air channels on Brazilian TV. It shows that six out of 10 UPP ads directed their abusive techniques towards children and adolescents, and almost all ads did not present adequate and clear information about the product. Further, it explored the main patterns in the use of those techniques in UPP ads, revealing that four out of five patterns were aimed at children or adolescents and that fast-food meals were associated with three of the five patterns identified. These findings emphasize the importance of protecting children from the exposure to unhealthy advertising as well as the urgent need to involve, educate and empower the judiciary branch so that more abusive advertising cases can be identified and judged. Finally, we reinforce the importance of applying robust, international NPM to substantiate and guide food and drink advertising regulations.

## FUNDING

This work was supported by the Coordenação de Aperfeiçoamento de Pessoal de Nível Superior (Capes) [finance code 001] and the Fundação de Amparo à Pesquisa (FAPEMIG), which provided Master’s degree scholarships during the research to [JSG]; and the International Development Research Centre (IDRC) [project ID—108166], which provided funding for the research.

## CONFLICT OF INTEREST

The authors declare that there are no conflicts of interest.

## ETHICAL STANDARDS DISCLOSURE

Not applicable, the study did not involve human subjects.
